# Continuous spinning aligned liquid crystal elastomer fibers with a 3D printer setup†

**DOI:** 10.1039/d1sm00432h

**Published:** 2021-05-03

**Authors:** Xueyan Lin, Mohand O. Saed, Eugene M. Terentjev

**Affiliations:** Cavendish Laboratory, University of Cambridge J. J. Thomson Avenue Cambridge CB3 0HE UK emt1000@cam.ac.uk

## Abstract

Fibrous liquid crystalline elastomers (LCE) are an attractive variant of LCE-based actuators due to their small thickness, leading to faster response times to stimuli, as well as the increased mechanical strength. Fabrication of LCE fibers has been attempted by various research groups using electro-spinning or micro-fluidic techniques, without much success. Here we propose an alternative way to achieve single-step continuous spinning LCE fibers in a more scalable and robust way, based on a liquid-ink 3D printer. We demonstrate this technique in our home-made device by dynamically extruding/stretching liquid crystalline oligomer mixed with photo-reactive cross-linker, to fix the aligned network under UV light after extrusion. The report also describes a protocol for material synthesis and identifies optimal conditions for the stable fiber spinning process. Microns-thick LCE fibers with two different compositions have been successfully spun, and demonstrated enhanced mechanical properties with the inherited thermal-actuation capability. This technique also demonstrates the potential to fine-tune the mechanical properties of fibers to enable further development in fiber-based LCE applications.

## Abstract 2

Fibrous liquid crystalline elastomers (LCE) are an attractive variant of LCE-based actuators due to their small thickness, leading to faster response times to stimuli, as well as the increased mechanical strength.

## Graphical abstract

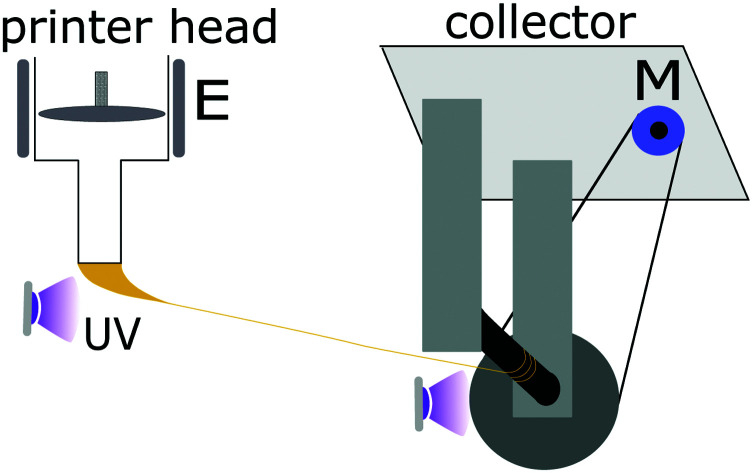

## 1 Introduction

Liquid crystalline elastomers (LCE) represent a unique type of soft material. One of their key features is that when manufactured in an aligned mono-domain state, they are capable of achieving large, reversible length changes, reflecting the equilibrium relation between the macroscopic shape of the soft solid and the internal degree of orientational order.^1^ Since the first development of LCEs in laboratory over 30 years ago,^2^ the two-step cross-linking process remains a standard fabrication scheme to produce mono-domain, aligned ‘single-crystal’ LCE.^3^ This process typically requires manual mechanical stretching of the initially loosely cross-linked LC gel, followed by the secondary cross-linking that fixes the induced orientational order, as well as sample shape. Such process is full of difficulties and is only practical in certain laboratory settings. Much effort is invested in searching for alternative methods to fabricate mono-domain LCE capable of reversible large-strain actuation in a fast and robust manner, in order to lay down the foundation for commercialization of their unique properties. However, currently most known methods apart from mechanical stretching, such as electromagnetic field^4^ and surface alignment^5,6^ are yet to solve the problem.

There are many reasons for the LCE fibers to be an attractive proposition for actuating systems. Once drawn and fixed by cross-linking, one may expect a higher degree of alignment in thin fibers.^7^ There is also a question of speed of actuation response: whether induced by ambient heating or by light, the macroscopic sample only responds once its whole body volume has been affected by the stimuli (*e.g.* the heat has propagated through). This is usually a rate-limiting factor in the resulting actuation. With thin fibers, however, both the heat and the order parameter diffusion throughout could take only milliseconds, in stark contrast to seconds and even minutes in more bulky LCE actuators. Therefore, we expect fibrous LCEs can react to stimuli faster owing to their miniature geometry. Such feature is much more promising in applications that require small-scale actuators of simple design. Such small-scale devices have been used as a laser beam steerer^8^ and in smart clothing.^9^ Moreover, if one considers the ‘Holy Grail’ of LCE acting as an actual artificial muscle, one recalls that that muscles themselves are built in fibrillar arrangement,^10^ and so using LCE in a form resembling the natural muscle fibers, accompanied with their faster response time, is promising.

Despite such promise, only few reports exist on realistically constructed actuators using LCE fibers. One of the reasons is the aforementioned difficulty of massively producing aligned, mono-domain bulk LCEs as the raw materials, let alone fibers. Since Naciri *et al.* manually pulled the very first LCE fiber and demonstrated its actuation in 2003,^11^ only a handful of groups have explored the melt-spinning,^7^ electrospinning,^12^ and microfluidic chips^13,14^ in attempts to mass-produce thin cross-linked LCE fibers. In fact, one paper reported an extraordinary actuation strain of an LCE fiber made in microfluidic stream,^14^ although the actuation by 2900% is certainly reflecting incomplete network crosslinking. However, microfluidic chip manufacturing is limited by its low throughput (millimetres per second), whereas LCE mats fabricated from electro-spinning suffer inherently from instability and random alignment, which then requires a complicated post-deposition alignment process. Consequently, a fast and convenient fabrication method of LCE fibers is urgently needed. Indeed, recently Roach *et al.*^15^ have used an eclectic method to extrude and stretch LCE into fibers, demonstrating interesting textile features that are enabled. However, their method requires multiple steps to align LCE fibers, whose diameter unfortunately still remains at millimetre scale.

**Fig. 1:**
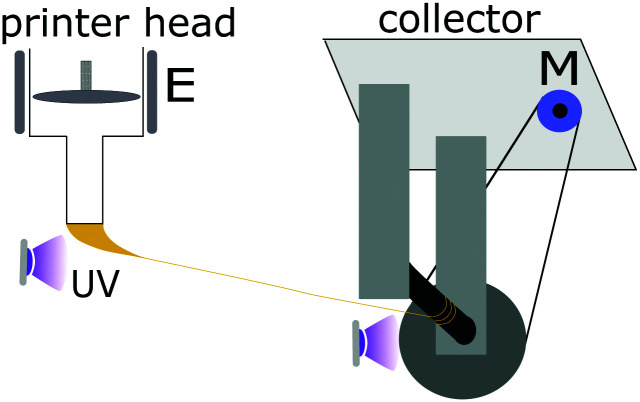
A proof-of-concept device for spinning LCE fibers, collecting the thread extruded from a 3D printer head onto a motor-controlled collector bobbin, with UV light completing the crosslinking in the fiber. E: extruder, M: motor.

Combining the concept of melt-spinning in conventional textile industries with the LCE two-steps cross-linking scheme, here we purpose a fast, scalable technique to spin and align micro-scale, straight LCE fibers. To do so, we utilize the previously reported thiol–acrylate/ene two-step “click” reaction^16–18^ to synthesize highly viscous liquid crystalline oligomer (LC oligomer) as extruding material. The oligomer is then extruded from a 3D printer nozzle in a way similar to that of melt spinning, only that UV cross-linking has now replaced solidification of melt polymer (Fig. 1). Using 3D printing in the “direct ink writing” (DIW) mode has been successfully used for alignment of nanostructures.^19–21^ Utilizing UV to crosslink the extruded filament *in situ* has been successfully demonstrated in various settings, including 3D-printed hydrogels fibers,^22^ and in microfluidic channels.^13,14^

A speed difference between the extrusion and the collector bobbin produces a stretched straight-flying LCE fiber with reduced diameter down to 40 μm. To demonstrate the versatility of our fiber spinning technique, we used two kinds of LCEs with different composition. One fiber is formulated with LCE crosslinked by conventional permanent covalent bonds,^18,23^ while the other is crosslinked by dynamic covalent bonds, capable of equilibrium bond-exchange.^24^ We then characterize the fibers and successfully obtain reversible actuation upon repeated heat–cooling cycles. These fibers also show a controllable variation of elastic modulus and strain-to-failure as the spinning conditions vary, and a high stress to break of several MPa. Surprisingly, we find that the degree of nematic alignment in our fibers is somewhat lower than in a traditional aligned monodomain LCE of nematic genesis, and the amplitude of their actuation stroke is slightly lower too (although still within 40–60%). We also confirm the great difference in actuation speed, the fibers responding with a full stroke within 1 second, while the LCE film takes at least a minute to fully change shape. We thus conclude that our purposed technique is able to fabricate aligned, strengthened LCE fibers, and is more readily scalable.

## 2 Experimental section

### Materials

Acrylate liquid crystal (LC) monomer (RM82, 95% purity) is purchased from Wilshire Technologies, Inc. Thiol spacer 2,2′-(ethylenedioxy)diethanethiol (EDDT, 95% purity), vinyl spacer 1,3-divinyltetramethyldisiloxane (DVS, 97% purity), vinyl cross-linker 1,3,5-triallyl-1,3,5-triazine-2,4,6(1*H*,3*H*,5*H*)-trione (TATATO, 98% purity) and 2,4,6,8-tetramethyl-2,4,6,8-tetravinylcyclotetrasiloxane (TVCS, 95% purity), Michael addition catalyst dipropylamine (DPA, 99% purity). We also compared using DPA with a more volatile catalyst triethylamine (TEA, 99% purity). The photo-initiator 2,2-dimethoxy-2-phenylacetophenone (I-651, 99% purity), radical scavenger butylated hydroxytoluene (BHT, 99% purity) are directly purchased from Sigma-Aldrich and used without further purification.

### Preparation of LC oligomer

We design a one-pot two-step thiol–acrylate/thiol–ene reaction strategy to prepare LC oligomers that can be drawn and crosslinkend into fibers with controllable polymer network topology (static or dynamic) from commercially available starting materials. We first prepare LC chains (oligomers) *via* the self-limiting thiol–acrylate Michael addition between a mesogenic diacrylate (RM82) and an isotropic dithiol (EDDT). The Michael addition was catalyzed *via* DPA. By controlling the molar ratio of thiol to acrylate, thiol-terminated oligomers were obtained. Next, after the fiber spinning process, the dithiol nematic oligomers were radically crosslinked with either vinyl siloxane crosslinker, (TVCS) and vinyl siloxane spacer, (DVS) by photo-initiated UV reaction to produce LCE fiber with dynamic polymer network topology (xLCE) or with regular vinyl crosslinker, (TATATO) to obtained LCE fiber with static polymer network topology. The overall reaction scheme is similar a previously reported method.^18,24^

For the dynamic xLCE network based on the siloxane bond exchange we use S-oligomer synthesized by mixing RM82, EDDT with additional DVS spacers and four-functional TVCS crosslinkers in a 25 ml vial without solvent, so that acrylate : thiol : vinyl becomes 1 : 1.2 : 0.2, counted by functional groups. In this way, DVS spacers and four-functional TVCS crosslinkers provide the equal number of vinyl reacting group. Since there is 0.1 molar fraction of each of DVS and TVCS, the crosslinking density becomes 10%. To obtain a conventional permanent LCE network we use **T-oligomer**, synthesized by mixing RM82, EDDT, and the three-functional TATATO crosslinker in a 25 ml vial without solvent,^18,25^ in the molar ratio of functional groups acrylate : thiol : vinyl = 1 : 1.25 : 0.25, respectively. This means that 25% crosslinking density was designed in this material. These ratios were chosen to optimally balance between 3D-printability, and the retention of the nematic order in the final material at room temperature. After that, 0.5 wt% BHT is added to increase the oligomer shelf-life. The reagents are carefully melt at 110 °C until no solid is present. After adding 1 wt% I-651 and 0.35 wt% DPA into the mixture at room temperature, it is vigorously mixed using vortex mixer, before degassed at 80 °C in vacuum chamber. Pour the degassed mixture into a stainless steel 3D printer syringe (Hyrel, KR-15) and cover the syringe at room temperature for the reaction to proceed. The highly viscous, fiber-forming oligomer should be synthesized after 30 minutes. In the thiol–acrylate–ene chemistry, the reaction of thiol–acrylate Michael addition proceeds much faster than thiol–vinyl reaction,^26^ which assures that the melt of the relatively short thiol-terminated oligomers can be used as a nematic liquid ink for 3D printing. The remaining thiol groups will react with vinyl cross-linkers (or additional chain extenders) on UV irradiation initiated by I-651.

The relatively low concentration of DPA catalyst (0.35 wt%) was empirically identified as optimal for a suitable catalytic speed and therefore ensures sufficient time for the degassed melt to be loaded into syringe. For the other test, *i.e.* calorimetry, we often used a higher concentration of DPA (1 wt%) to speed up this reaction, since here we were not concerned about the trapping of air inside the oligomer melt.

### Fiber spinning setup

A home-made extrusion set-up is used to spin LCE into fibers using the reactive oligomers. The oligomer is initially transferred into a syringe (KR2, Hyrel3D), which was then locked and loaded into a 3D printing system (Engine HR, Hyrel3D). The oligomer temperature is maintained at 40 °C in order to facilitate material flow, while retaining ink nematic phase. The oligomer is then extruded through the nozzle (with 400 μm diameter) by a motorized piston at a constant flow rate of about 4 μl min^−1^. The extruded oligomer needs to be partially cross-linked by 365 nm UV light immediately on the nozzle exit, which is achieved by exposing the vicinity of the syringe nozzle to an appropriate amount of UV light (365 nm UV LED, Opulent Americas, providing the flux of about 3 mW cm^−2^). As the material gels on this initial UV exposure, we use tweezers to manually pull the extruded oligomer away from the nozzle, forming a straight fiber, and land it onto a plastic bobbin rotating at a constant speed, see Fig. 1. The fiber wraps around the collector bobbin, and cured finally with an another 365 nm UV LED located nearby. The difference in bobbin rotation speed controls the stretching, and thus the fiber thickness.

### Preparation of the monodomain LCE control

#### Bulk xLCE based on S-oligomer (SM)

Mix the prescribed ratio of EDDT, DVS and TVCS in a vial with 0.1 wt% of I-561. Expose the vial to 365 nm UV light while shaking vigorously for 5 minutes to cap the siloxane units with thiol. After that, the corresponding amount of RM82 and 60 wt% toluene is added into the vial, along with 1 wt% DMAP solution (dissolved in minimal amount of acetonitrile). Vortex the mixture and use necessary amount of heat to dissolve all reagents. Then, 1 wt% of DPA is added to facilitate the thiol–ene reaction. The solution of reagents is degassed under vacuum and injected into a glass mould, and cured overnight at 60 °C. After evaporating solvent from the as-synthesized poly-domain LCE film, a rectangular strip is cut out from it to make into mono-domain LCE. The strip is attached to a free-hanging weight while being heated to 200 °C for at least 2 h under tension to initiate the bond exchange and plastic flow.^24^

#### Bulk LCE based on T-oligomer (TM)

Mix the prescribed ratio of all chemicals used when making T-oligomer in a vial and allow the reaction to proceed without addition of solvent. The viscous oligomer is taken out after 30 minutes of reaction and exposed to weak UV light (0.5 mW cm^−2^) to establish the initial crosslinking. During this time the oligomer experiences transition from fluid to gel. Within this time window, the material is slowly stretched uni-axially, by hand, usually to a strain greater than 200%, until a uniform thin strip is obtained. The aligned LCE strip is further cured under stronger UV light for an additional 5 minutes.

### Differential scanning calorimetry (DSC)

We investigate the nematic–isotropic transition (*T*_ni_) using the DSC4000 instrument from PerkinElmer. The heating and cooling rate was 10° min^−1^, and results were taken on the final heating runs. In order to understand the influence of different impurities to the observed transition temperature, we have intentionally added in solvent or extra catalyst in the beginning of the click reaction, as discussed in the main text.

### Polarised optical microscopy (POM)

Olympus BX41 was used to acquire images of the spun LCE fibers. Using cross-polarizers to enhance the contrast of images, the alignment of nematic director in spun fiber is observed by rotating the angle between cross-polarizers and sample axis from 0° to 45°. ImageJ software is used to measure the average fiber diameters from the images. The average cross-section area of a single fiber is then calculated and recorded.

### Wide angle X-ray scattering

We determined the uniaxial (nematic) orientational parameter 
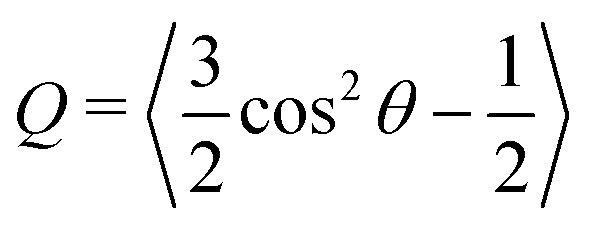
 from the azimuthal intensity distribution in a wide-angle X-ray scattering map, obtained on a Philips PW1830 generator with the wavelength of 0.154 nm, with a wide-area CCD detector from Photonic Sciences Ltd. The monodomain bulk film, or the bundle of parallel fibers, were suspended in front of the beam exit, and the intensity collected from the ‘nematic’ reflection at the characteristic length of 4.8 Å. The order parameter was calculated with the Maier–Saupe mean-field orientational distribution *P*(*θ*) ∝ exp[*J* cos^2^ *θ*/*k*_B_*T*], obtained by fitting to the scattering data.

### Stress–strain curve of a single fiber

We use a home-made device to perform tensile testing of the spun fibers, and compare the result with their bulk LCE counterparts. In such tensile stress–strain tests, the strain rate was kept at 0.01 s^−1^. Because the stress response of a single, untwisted fiber is too low to be measured reliably in our device, we measured it through averaging the stress of the compact fiber bundles cut from a bobbin. The stress of a single fiber is then obtained by dividing the measured tension with the total cross-section area of the bundle (separately knowing the number of fibers in the bundle, calculated based on the spinning time and collector's rotation speed, and their diameter found in optical microscope).

### Dynamic mechanical analysis (DMA)

Repeated cycles of thermal actuation of fiber samples were measured and recorded using DMA850, from TA. The heating/cooling rate was kept at 5 °C min^−1^, and the temperature was swept in linear cycles from −20 °C to 120 °C and back. The maximal actuation strains of each samples are calculated from the cooling run, as their isotropic lengths are assigned as the initial length.

## 3 Results and discussion

**Fig. 2:**
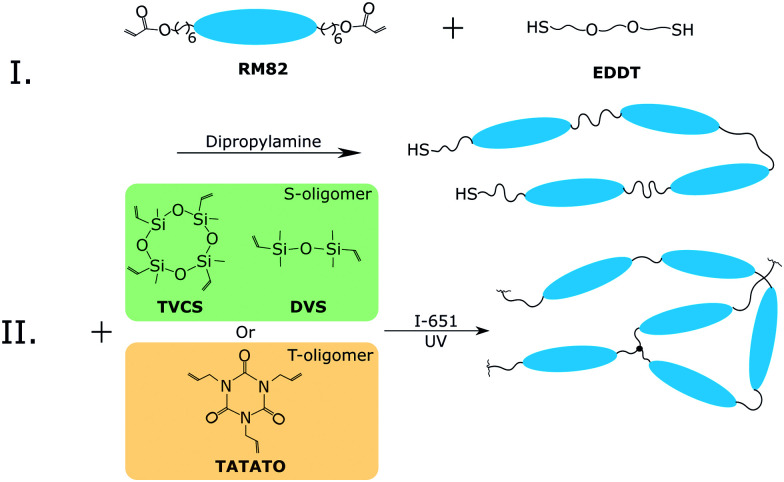
Scheme of reactions, illustrating the two distinct steps. Step I is done in a glass vial, the resulting thiol-terminated oligomer fluid is added into the syringe. Step II occurs after extrusion, when UV-irradiation achieves the crosslinking of fibers in their aligned state, and consolidates this crosslinking by further illuminating on the rotating collector bobbin.

The purpose of this study is to fabricate strong, well-aligned, and actuating LCE fibers using a home-made fiber drawing setup, where the fibers can be made either from conventional permanent or dynamic liquid crystalline elastomer networks. The xLCE fibers were synthesized following previously reported reaction methods by Saed *et al.*^27^ We use a two-step thiol–acrylate/ene reaction scheme (Fig. 2). Firstly, the Michael addition of thiol–acrylate was catalyzed by DPA. By controlling the molar ratio of thiol to acrylate, thiol-terminated oligomers were obtained. Next, after the fiber spinning process, the thiol-capped nematic oligomers were radically crosslinked with vinyl siloxane crosslinker, (TVCS) and vinyl siloxane spacer, (DVS) by photo-initiated reaction to produce xLCE fiber with the dynamic polymer network topology. The regular vinyl crosslinker, (TATATO) was used to obtain LCE fibers with the permanent polymer network topology. Upon completion of the oligomerization reaction, the material flows much like a melt of thermoplastic, which can be easily pulled into thin fibers by hand, and UV-crosslinked in the same way as the xLCE system.

However, the actual fiber-forming capability of oligomer relies on it having a sufficient degree of polymerization (molecular weight) before extrusion and crosslinking. A viscous high molecular weight liquid can easily be drawn into fiber, whereas oligomers with low molecular weight do not form stable fibers (due to the surface tension). Informed by our recent work on correlating the rheological properties and the mesogenic behavior (*e.g.* nematic to isotropic phase transition temperature) to the degree of polymerization and molecular weight of the oligomer,^24^ we have designed oligomers with molar ratio of acrylate : thiol = 1 : 1.2 to have the average molecular weight between 7000 to 10 000 g mol^−1^ for T-oligomer and S-oligomer, respectively. Oligomers with molecular weight in this range can form extrude-able materials, and are readily pulled into fibers. Note that prolonged exposure of ambient light and storing above room temperature could induce some of crosslinking within the oligomer melt, which makes its rheological characteristics sub-optimal for fiber drawing.

**Fig. 3:**
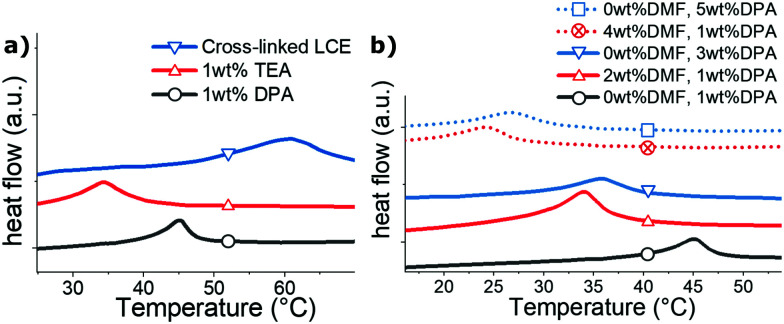
Differential calorimetry study of nematic transitions, all data in heating at 10° min^−1^. (a) The *T*_ni_ of various oligomers. Using undiluted 0.35 wt% DPA catalyst, repeatable *T*_ni_ of *ca.* 45 °C can be obtained. On the other hand, volatile TEA as catalyst can easily evaporate in the degassing step, which leads to incomplete reaction and therefore shorter chain-lengths and lower *T*_ni_, even when the oligomer was left to react for 24 h. Such poorer-reacted oligomer cannot be pulled into stable fibers. When we cross-link long oligomer chains, the *T*_ni_ becomes higher than 50 °C, an observation also found in premature gelled oligomer. (b) Adding isotropic impurity to the oligomer also decreases its transition temperature. This is illustrated by increasing concentration of solvent DMF plus catalyst DPA, from 1 wt% to 3 wt%, to 5 wt%.

Apart from looking at the rheology, it is known that the nematic–isotropic transition temperature *T*_ni_ increases with liquid crystalline polymer chain-length.^28^ We hence utilize DSC as a coarse quality control of the fiber-forming ability in our synthesized oligomer ink. As the thiol–acrylate chain-extending reaction progresses, longer oligomer chain length leads to rising of the *T*_ni_, which finally saturated around a certain temperature (*e.g.* 45 °C for T-oligomer) when the reaction reached its completion. The saturated oligomer fluid contains sufficiently long chains, which can be easily pulled into fibers by hand. We noticed, however, if a full crosslinking is implemented to the oligomer, the final elastomer reaches *T*_ni_ = 60 °C, see Fig. 3a. Hence a transition temperature above 45 °C often suggest the occurrence of premature crosslinking within the oligomer due to the above-mentioned reasons. A lower transition temperature, on the other hand, indicates an incomplete thiol–acrylate Michael chain-extending reaction, which is often found in system catalyzed with a more volatile catalyst, such as TEA, also shown in see Fig. 3a.

We keep the entire synthesis solvent-free, mainly because any unnecessary isotropic impurity (*e.g.* conventional solvents that remained in the oligomer) is detrimental to the mesogenic power and results in lower *T*_ni_ and poor fiber-forming ability. Fig. 3b illustrates this effect of increasing impurity on lowering *T*_ni_; in both plots (a) and (b) the black curve corresponds to the oligomer used in our fiber production, but several other materials are illustrated: with a total of 3 wt% and 5 wt% of impurity achieved in different ways. For these reasons, we work with no solvent and the lowest possible concentration of catalyst.

The most important parameter for a sustained LCE spinning process (continuous spinning for at least 10 minutes) is the intensity of UV light, particularly the intensity near syringe nozzle, see Fig. 1. On the one hand, too-weak light intensity does not bestow enough cross-linking points to the stretched oligomer ink in a short time, so the fiber would eventually break by tapering off while travelling to the collector. On the other hand, too-strong UV light, while preventing the fiber breaking in flight, may cause immediate strong gelling of the extruded oligomer filament before alignment, and causes jamming the nozzle. The appropriate UV intensity in our experiments was around 3 mW cm^−2^, but it remained rather case-specific depending on the oligomer temperature, initiator concentration, collector rotation speed, and the extrusion flow rate. A faster collector speed and higher flow rate combination requires the higher UV intensity near the nozzle in order to maintain fiber integrity and ensure stable spinning, and *vice versa*.

**Fig. 4:**
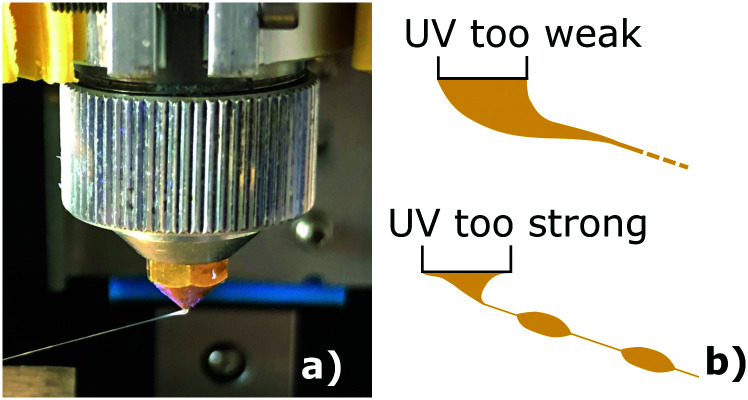
(a) A stable size lump on the nozzle exit is associated with stable spinning. (b) When UV intensity is not suitable, the lump changes its size, indicating various instability modes.

In practice, a suitable UV intensity for a specific profile of collector speed and flow rate is typically associated with the observation of the extruded oligomer forming a stable-sized lump (Fig. 4 right below the nozzle exit). This interesting feature can be conveniently monitored to indicate UV abnormality, hence the overall spinning stability. As an example of unstable spinning under a constant flow rate (Fig. 4b), a fast collector speed with UV intensity weaker than ideal is likely to enlarge and elongate the lump, where the uncrosslinked oligomer filament tapers off at the tip of the lump. On the other hand, a slow collector speed accompanied by a very strong UV intensity tends to crosslink the lump into plugs, before it is stretched and aligned into the fiber. In both situations, the size-change of the lump foretells instability of spinning, and in the worst case imminent breaking of the flying fiber. Under a suitable light intensity, the lump size remains unchanged and the fiber quickly accelerates toward the collector. At the same time, the ink filament is stretched by the speed difference and reduces its diameter, creating a straight, thin fiber in the air. The fiber is then wrapped by the collector bobbin and further cured with the other UV source that operates at the maximal intensity around the bobbin, see in supplementary video 1.†

**Fig. 5:**
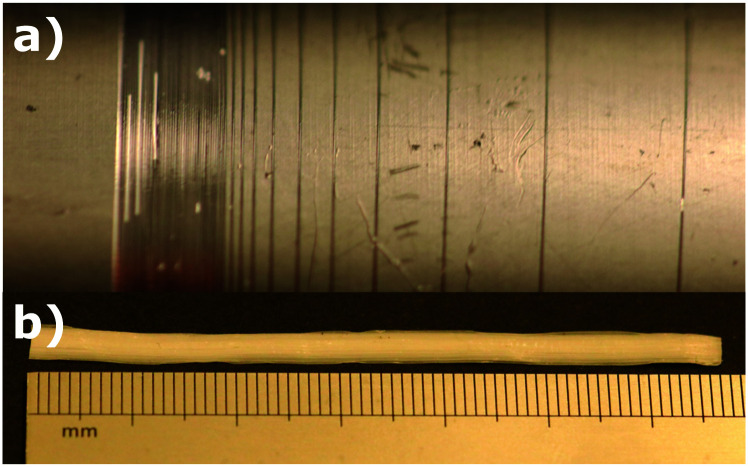
(a) The spun LCE fiber made from S-oligomer collected on a plastic cylinder. (b) The compact fiber bundle is cut out from the roll, and used for subsequent characterization.

**Fig. 6:**
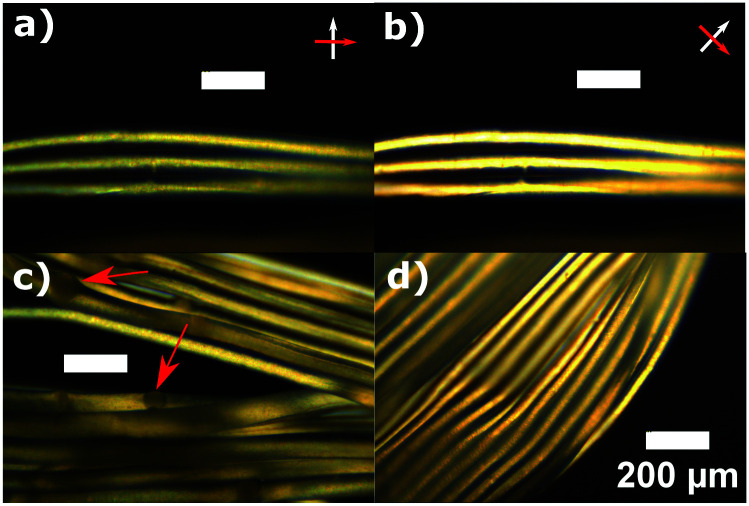
LCE fibers made from S-oligomer examined under crossed polars in optical microscopy. (a and b) Change of brightness between parallel and 45° alignment of polars indicates mesogen alignment within the fibers. There is no complete extinction because of the round shape of a fiber, making a lens effect in the center. (c) Semi-crystalline spheres (indicated by arrows) are seen in the fibers, which we attribute to the phase-separation between oligomer and siloxane-based cross-linker. These reversible crystals start to appear after storing the oligomer for more than 1 day before spinning. (d) Annealing the oligomer above its isotropic temperature before spinning eliminates such inhomogeneity.

Fig. 5 shows the fibers collected using the technique described above. The fiber is collected into a roll on the bobbin surface, and we sometimes cut out a bundle from the roll and use it in order to magnify the forces in fiber tensile strength measurements. The number of fibers in such a bundle is known from the number of rotations of the bobbin within the collection time. To obtain the average diameter of the spun fiber bundles, samples are examined under POM (Fig. 6). In the microscopy, we observed a difference in brightness when rotating analyzer/polarizer pair from 0° to 45° away from the fibers main axis, indicating the fibers are crosslinked with a strong uniaxial alignment.

**Fig. 7:**
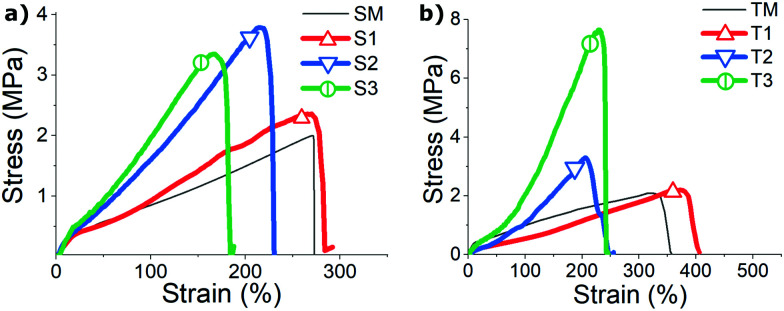
(a) Stress–strain curves scaled to represent of a single S-oligomer fiber (samples labelled in the plot), compared with the non-aligned poly-domain LCE film of the same composition (dashed line). (b) Stress–strain curves of a single T-oligomer fiber, compared with the corresponding aligned mono-domain LCE film (dashed line).

The spun fibers show enhanced mechanical properties above certain spinning speed (higher modulus, stress to break, and ductility) compared to bulk mono-domain samples made with the same compositions (Fig. 7). We found the stress response of both bulk (SM and TM) samples similar to that of S1 and T1 fibers, suggesting that under our slowest spinning speed no significant enhancement of strength is endowed to the fibers. In contrast, S2–S3 and T2–T3 fibers, in which samples were spun with a higher speed, did exhibit greater breaking stress and increasingly higher tensile modulus. Naturally, this is expected from polymer fiber spun with smaller diameters (which in turn is determined by the fiber spinning conditions). Table 1 qualitatively compares the diameter of six fiber samples, and their measured order parameters. Additional order parameters from the two bulk mono-domain control samples are also listed in the table. In general, all fibers (T1–T3 and S1–S3 samples) have an expected decreasing trend in fiber diameters, and an unexpected decreasing trend in their nematic order parameters, as the spinning speed increases. We rationalize this that the reduced diameter introduces the much higher surface-to-volume ratio to enhance the stabilizing effect of fiber surface, and also likely more intense chain entanglement, both enhancing internal constraints for the nematic order.

**Table 1:** Table of sample codes and parameters. T-oligomer and S-oligomer materials (different in their crosslinking agent, and chain extenders) are extruded with a same flow rate 7 μl min^−1^ and same nozzle size (0.4 mm). Mono-domain control samples for each composition are labelled as TM and SM

Sample	Oligomer	Collector speed (mm s^−1^)	Fiber diameter (μm)	Order parameter
T1	T-oligomer	15	82	0.49
T2	T-oligomer	45	50	0.45
T3	T-oligomer	75	43	0.45
TM	T-LCE	—	—	0.60

S1	S-oligomer	15	87	0.66
S2	S-oligomer	45	49	0.65
S3	S-oligomer	75	40	0.58
SM	S-xLCE	—	—	0.58

Fig. 7 also shows that all materials have a considerable stored elastic energy density (determined as roughly a half of stress × strain at break): between approximately 2.7 × 10^6^ J m^−3^ ≈ 2.7 J g^−1^ and 10^7^ J m^−3^ ≈ 10 J g^−1^. It is interesting to compare this with the latent heat of the nematic–isotropic transition, which is only *ca.* 0.2 J g^−1^. It is also clear that thinner fibers (*cf.*Table 1) have a higher elastic modulus, but lower strain to break, albeit still in many hundreds of percent.

**Fig. 8:**
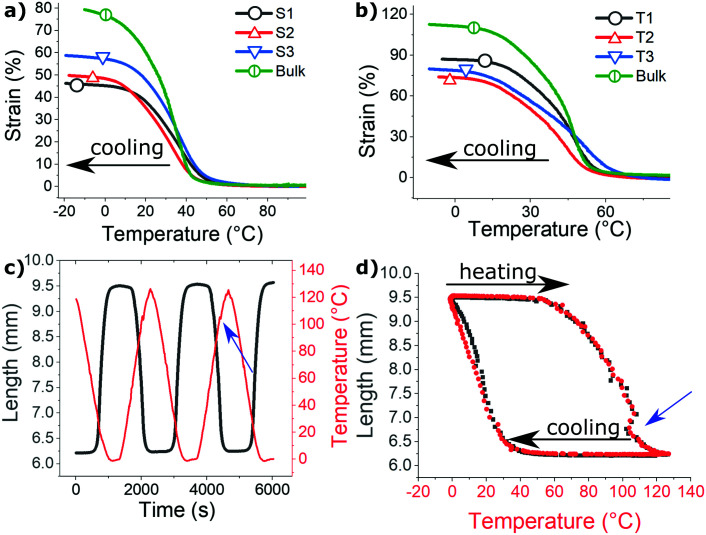
Thermal actuation of the bundles under a constant load of 30 kPa, at a relatively high rate of 5 °C min^−1^. (a and b) Different bundled samples, as labelled in the plots, on cooling. In both plots, the curve labelled by a circle is the actuation stroke of an aligned mono-domain film of the corresponding material, in both cases having a slightly higher amplitude than the fiber actuation. (c and d) Detailed data from the S3 bundle, showing repeated actuation cycles in two representations (*vs.* time and *vs.* temperature). The artefact marked with blue arrows is discussed in the text.

Apart from their enhanced mechanical strength, the spun fibers are all able to thermally actuate, with a much faster response due to the fast heat diffusion across thin material. Fig. 8 illustrates this actuation in almost load-free conditions (we did maintain a low tensile stress of 30 kPa to keep the fiber bundles straight, although the fibers could withstand several MPa of stress as illustrated in Fig. 7). Plots (a) and (b) compare the actuation stroke in S- and T-fibers, cooled from their isotropic phase, with their bulk monodomain control. The fibers generally show a slightly lower amplitude of actuation stroke, compared to their well-aligned bulk monodomain counterparts (and also a more diffused transition). This may be surprising, as one might have expected that strongly drawn fibers would have higher alignment, and therefore, higher actuation range. In fact, earlier publications on LCE fibers frequently reported a much increased actuation range.^7,14^ However, the order-parameter data in Table 1, obtained by X-ray characterization, does not support the assumption that a drawn fiber has better alignment, at least in our 3D-printing process. Also, considering how much more mechanical strength the fibers have (Fig. 7), it is clear that the fiber surface provides a lot of stabilization for the polymer network. As a result, the range of their actuation stroke is consistently diminished, albeit still within the same order of magnitude as the ‘native’ bulk LCE actuation.

We find no systematic difference in the actuation stroke between the fibers in each material. This comparison of low-free thermal actuation implies that the nematic director is fully aligned during fiber drawing, even at the lowest pulling speed of the collector (in S1 and T1). We have to conclude that the strong shear flow in the fibers, before and during their crosslinking, creates a sufficient number of internal constraints and defects. That is, most of the nematic alignment was already established by the shear forces during extrusion from the nozzle, and fixed already by the first UV source.

Plots (c) and (d) in Fig. 8 show the multiple cycles of driven actuation and their time-dependence. The cycles are highly reproducible (plot (c) only shows three cycles for clarity), but the plot (d) also highlights a large thermal hysteresis that occurs due to a high rate of forced heating/cooling. It is interesting to note a characteristic feature in these plots, obtained in a (DMA) device that dynamically heats the sample attempting to maintain the prescribed rate of temperature change in a small chamber. When the sample undergoes the nematic–isotropic transition, some of this heat is absorbed as latent heat of this 1st order phase transition, and the instrument does takes a bit of time to adjust to that – the actual measured temperature drops momentarily creating a feature in the cycled plots.

**Fig. 9:**
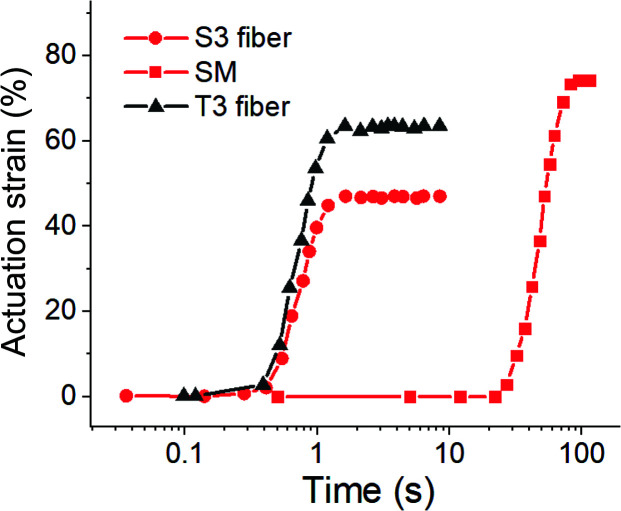
The comparison of actuation speed in 40 μm-thick S3 and T3 fibers, and in a bulk SM monodomain film of thickness 0.6 mm. The *t* = 0 in the plot is the moment of time when the infrared heating lamp is removed, and the natural cooling of load-free samples in ambient air produces spontaneous elongation.

The important characteristics of thermal (as well as photo-induced) actuation in LCE is the speed of the response. One of the main reasons for us to investigate thin LCE fibers was to assure that heat exchange is very fast across the sample, and the resulting rate of actuation much higher than in corresponding bulk samples. Fig. 9 illustrates this point by comparing the natural load-free elongation of aligned LCE samples on cooling from isotropic phase. In these tests, we freely hang the LCE fibers and the aligned mono-domain LCE film (applying a very small weight to keep the samples straight), and allow them to naturally cool in the ambient air on the bench – while taking video (see supplementary video 2†), and later analyzing its frames for the time evolution. Here we used the powerful incandescent lamp to heat the samples by infrared irradiation, so the moment the lamp was turned away was easy to identify as the origin of time in the test. Both 40 μm-thick fibers take less than 1 second to complete the whole actuation stroke. In contrast, a more bulky film takes over 20 seconds to cool down to the actuation range, and then the actuation stroke takes over a minute of time. As in Fig. 8, we find that the range of actuation is slightly higher in the mono-domain aligned film, compared to the fibers.

**Fig. 10:**
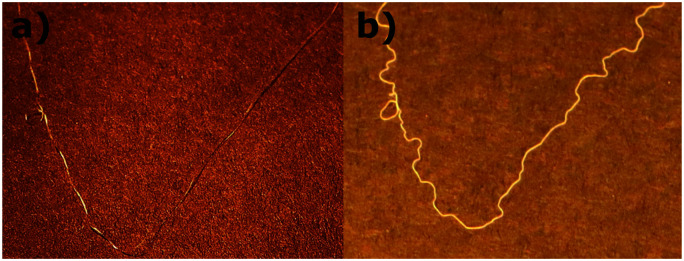
Observing the reversible change in natural length of a free-standing fiber: (a) in the isotropic phase at elevated temperature. (b) In the nematic phase at room temperature.

Finally, Fig. 10 illustrates the natural change in the length of free-standing fiber on heating and cooling. As the filament rests on a flat substrate, its elongation in the nematic phase causes its shape to curl between several random points of attachment to the substrate.

## 4 Conclusions

In conclusion, here we proposed a simple technique to produce continuous micron-scale, aligned LCE fibers that feature typical reversible thermal actuation. We described the detailed steps in the synthesis and the fiber spinning process, and highlighted key precautions. We tested the spun fibers under tensile stress and found their modulus as well as ultimate breaking strength are enhanced compared to the corresponding aligned bulk samples, which is rendered possible by spinning LCE into fibrous shapes. The surprising result was that the internal alignment, and the actuation range in the fibers was slightly lower than in their bulk counterparts, which we understand as a consequence of the oligomer extrusion and photo-crosslinking process. As expected, the speed of the LCE actuation response is much greater in the thin fibers, which is the main attraction in using fibrous actuators. We think that the purposed fabrication scheme is generic to other photo-crosslinkable LCE compositions, and this opens up opportunities for mass production of aligned LCE fibers, which facilitate the commercialization of LCE-based actuators.

## Conflicts of interest

There are no conflicts to declare.

## Supplementary Material

SM-017-D1SM00432H-s001

SM-017-D1SM00432H-s002
